# Effects of Finerenone on Cardiovascular and Chronic Kidney Diseases: A New Weapon against Cardiorenal Morbidity and Mortality—A Comprehensive Review

**DOI:** 10.3390/jcdd10060236

**Published:** 2023-05-28

**Authors:** Francesco Piccirillo, Paola Liporace, Annunziata Nusca, Vincenzo Nafisio, Andrea Corlianò, Francesca Magarò, Raffaele Antonelli Incalzi, Gian Paolo Ussia, Francesco Grigioni

**Affiliations:** 1 Fondazione Policlinico Universitario Campus Bio-Medico, Via Alvaro del Portillo, 200, 00128 Roma, Italy; f.piccirillo@unicampus.it (F.P.); paola.liporace@unicampus.it (P.L.); vincenzo.nafisio@unicampus.it (V.N.); andrea.corliano@unicampus.it (A.C.); f.magaro@unicampus.it (F.M.); r.antonelli@policlinicocampus.it (R.A.I.); g.ussia@policlinicocampus.it (G.P.U.); f.grigioni@policlinicocampus.it (F.G.); 2 Research Unit of Cardiovascular Sciences, Department of Medicine and Surgery, Università Campus Bio-Medico di Roma, Via Alvaro del Portillo, 21, 00128 Roma, Italy; 3 Research Unit of Geriatrics, Department of Medicine and Surgery, Università Campus Bio-Medico di Roma, Via Alvaro del Portillo, 21, 00128 Roma, Italy

**Keywords:** chronic kidney disease, cardiovascular disease, type 2 diabetes, mineralocorticoid receptor, mineralocorticoid receptor antagonist, finerenone

## Abstract

Patients with cardiovascular disease (CVD) and chronic kidney disease (CKD) show high rates of cardiorenal outcomes. In addition, the progression towards renal failure and cardiovascular events rises as CKD worsens. Several studies suggest that the activation of the mineralocorticoid receptor (MR) induces cardiac and renal injury, including inflammation and fibrosis. Finerenone is a novel, nonsteroidal, selective MR antagonist (MRA) which has demonstrated anti-inflammatory and anti-fibrotic effects in pre-clinical studies. Moreover, two large trials (FIDELIO-DKD and FIGARO-DKD) investigated the renal and cardiovascular outcomes in patients with mild to severe CKD in type 2 diabetes which received finerenone. On these bases, this comprehensive review aims to summarize the current knowledge regarding finerenone and its effects on CKD and the cardiovascular system, emphasizing its role in modifying cardiorenal outcomes.

## 1. Introduction

Cardiovascular disease (CVD) and chronic kidney disease (CKD) are closely connected since they share common risk factors and pathophysiological pathways and influence mutual evolution. Indeed, a reduced cardiac performance, such as in a heart failure (HF) setting, affects renal functions through an activated neurohormonal and inflammatory cascade, and increases the venous pressure and hypoperfusion of the kidneys. In contrast, CKD impacts cardiovascular functions by inducing hypertension and vascular calcification [1,2,3]. In patients with CKD requiring dialysis, CVD is recognized as the leading cause of death [4], whereas CKD is an independent predictor of mortality and morbidity in patients with HF [5].

Therefore, the turning point in the prognosis and long-term management of CVD and CKD may be the early use of drugs that act simultaneously on the heart and kidney. Particularly, a treatment with sacubitril/valsartan leads to a slower decline in renal functions and improves cardiovascular outcomes in patients with HF [6]. However, the optimal titration of this combination is often hampered by suboptimal creatinine levels, as only few studies included patients with an estimated glomerular filtration rate (eGFR) < 30 mL/min [7]. Similarly, the use of sodium–glucose transporter 2 inhibitors (SGLT2i) for the treatment of heart failure also offers renal protection [8,9]. A recent study in patients with advanced CKD (eGFR 25–45 mL/min) showed that empagliflozin was associated with a reduced risk of renal progression or cardiovascular death compared to a placebo (hazard ratio, 0.72; 95% confidence interval [CI], 0.64–0.82; *p* < 0.001) [10]. Despite this, cardiovascular outcomes are generally worse in patients with CKD than in those with normal renal functions. It should be noted that patients with impaired renal functions cannot benefit from optimized cardiovascular medical therapy, as there are no studies supporting its prescription in this setting.

The renin-angiotensin-aldosterone system (RAAS) is the direct link between the heart and the kidneys and is one of the main mechanisms for the homeostasis of sodium, volume, osmolarity, renal blood flow, and blood pressure [11]. Studies have shown that the activation of the mineralocorticoid receptor (MR) induces cardiac and renal injury, including inflammation and fibrosis [12,13]. In this context, mineralocorticoid receptor antagonists (MRAs) were developed and are now widely used in the treatment of HF, refractory hypertension, and various renal diseases [5]. They work by inhibiting the action of aldosterone on its receptor. This results in a reduction in cardiac remodeling, a reduction in inflammation, and a reduction in proteinuria [14,15]. Finerenone is a novel non-steroidal selective MRA with a stronger mineralocorticoid receptor binding potential than eplerenone and spironolactone [12,16]. Finerenone has been shown to have anti-inflammatory and anti-fibrotic effects in preclinical studies [16]. It has also been shown to improve cardiorenal outcomes in patients with type 2 diabetes mellitus (DM) and mild to severe CKD (FI-DELIO-DKD and FIGARO-DKD clinical trials) [17,18].

Due to these promising findings and meaningful clinical implications, this comprehensive review aims to summarize the pharmacokinetic and pharmacodynamic properties of this novel drug, focusing on its potential for modifying cardiorenal outcomes.

## 2. Methods

This comprehensive review was conducted via a literature search of the PubMed, EBSCO Embase, and Cochrane databases of systematic reviews up to February 2023, using the following MESH terms and keywords in various combinations: “finerenone”, “chronic kidney disease”, “cardiovascular disease”, “type 2 diabetes”, “cardio-renal system”, “mineral-ocorticoid receptor”, “mineralocorticoid receptor antagonists”, “steroidal mineralocorti-coid receptor antagonists”, and “non-steroidal mineralocorticoid receptor antagonists”. As a comprehensive and not systematic review, we had no predetermined research questions or specified protocols. We limited the search to articles published in English in high-impact journals between January 2010 and January 2023, including review articles and clinical and pre-clinical studies. High-impact journals were included in case of an impact factor superior to 3.0. The first part of this review analyzes the association between CKD and CVD; the second section summarizes the RAAS system, the mineralocorticoid receptor, and the MRAs; and the final section focuses on finerenone, the main subject of the review.

More details regarding research methods are described in Appendix A.

## 3. Chronic Kidney Disease and Cardiovascular Disease: A Dangerous Association

**Figure 1:**
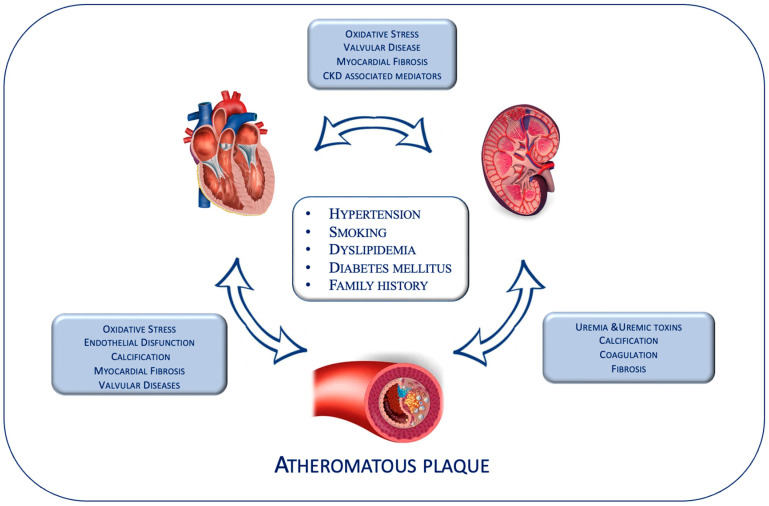
Correlation between chronic kidney disease and cardiovascular disease.

Approx. 50% of all patients with advanced CKD suffer from cardiovascular disease, and CV events are responsible for approx. 40–50% of all deaths in patients with stage 4 to 5 CKD [19,20]. The cause of this association could be explained by the presence of shared risk factors between CKD and CVD, such as hypertension, smoking, dyslipidemia, diabetes mellitus, and family history (Figure 1) [19]. Firstly, patients with CKD show significant lipid profile modifications, consisting of hypertriglyceridemia and low high-density lipoprotein (HDL) cholesterol levels [21]. In addition, in the CKD setting, the presence of uremia and uremic toxins, a pro-inflammatory environment, and raised oxidative stress could alter the composition of HDL proteome and lipidome, inducing a more atherogenic profile [22,23]. Moreover, hypertension and hyperglycemia, even in the absence of overt diabetes, represent two strong risk factors for developing and progressing CVD, CKD, and subsequent multi-district vascular diseases [24,25]. Beyond traditional risk factors, several uremic-related metabolic alterations are also involved in the increased CV risk in patients with CKD, such as renal anemia, secondary hyperparathyroidism, oxidative stress, endothelial dysfunction, and mineral metabolism disorders [26].

In this regard, many studies established an association between CKD and CVD. Particularly, Go et al. showed a correlation between the reduced GFR and the risk of death, cardiovascular events, and hospitalization [27]. Similarly, Manjunath et al. obtained similar results in an elderly population [28]. Moreover, two clinical trials, the Heart Outcomes and Prevention Evaluation (HOPE) study and the Hypertension Optimal Treatment (HOT) study, showed a meaningful increase in cardiovascular events with higher serum creatinine values [29,30].

In addition, several studies exhibited an association between CKD and coronary artery calcifications (CACs), a well-known risk factor for CVD [31,32]. The high CAC burden observed in CKD patients was related to a bone mineral metabolism disorder that determined high blood calcium and phosphorous levels leading to vascular calcification [33,34]. Furthermore, the patients with severe CKD often show left ventricle (LV) hypertrophy, as approx. 50% of patients with eGFR < 30 mL/min could reveal LV hypertrophy [35]. Two principal mechanisms could induce LV hypertrophy in these patients: a volume overload that causes eccentric hypertrophy and a pressure overload inducing concentric hypertrophy [36]. Indeed, in the CKD subset, the reduced glomerular filtration translates into a lower renal excretion of sodium with a consequent volume overload and activation of the renin-angiotensin-aldosterone system, with a subsequent hypertension and pressure overload [37]. In addition, reduced erythropoietin production with chronic secondary anemia is another cause of volume overload [35].

Lastly, LV hypertrophy in CKD is also secondary to increased vascular stiffness and calcification, which produce a raised afterload and cardiac workload and a reduced coronary artery perfusion pressure, leading to myocardial hypertrophy and microvascular ischemia [38]. In addition, renal failure is characterized by an inappropriate hyper-activation of the sympathetic system, including the reflex and neurohumoral pathways, which is already evident at the earliest clinical stage of CKD and directly related to the progression of renal failure, which is most pronounced in the end-stage phases [39]. In the end, the presence of LV hypertrophy with consequent ischemia due to the oxygen demand–supply mismatch, the high burden of CACs, the electrolyte imbalance, a sympathetic over-activation, and the alteration in drugs concentrations could induce an increased risk of sudden cardiac death in patients with CKD [40].

## 4. Renin-Angiotensin-Aldosterone System and Mineralocorticoid Receptor Antagonists (MRAs)

The renin-angiotensin-aldosterone system (RAAS) plays a crucial role in the regulation of the electrolyte balance, blood pressure, and fluid homeostasis [41]. Renin is secreted by the granular cells of the juxtaglomerular apparatus of the kidney. Its secretion is stimulated by the sympathetic nervous system, the reduced sodium content of the distal convoluted tubule, and the reduced perfusion pressure in the kidney [11]. Renin hydrolyses angiotensinogen, a protein secreted by the liver, to Angiotensin I, which in turn is converted into Angiotensin II by the activity of an angiotensin converting enzyme (ACE) [42]. Angiotensin II has a direct effect on vasoconstriction and stimulates the release of aldosterone from the adrenal cortex [43]. Aldosterone binds the mineralocorticoid receptor (MR) located in the distal tubules and collector ducts, inducing increased sodium reabsorption and potassium secretion through an increased concentration and activity of the epithelial sodium channels and the Na^+^/K^+^ ATPase pump [43]. The mineralocorticoid receptor is a member of the nuclear hormone receptors, a group of receptors which, upon the binding of their ligand, act as transcription factors and play a crucial role in the pathogenesis of cardiovascular and renal diseases. Indeed, the mineralocorticoid receptor is expressed not only in the epithelial cells of the distal nephron, but also in cardiomyocytes and cardiac fibroblasts, where it could directly stimulate the proliferation of these cells, promoting inflammation, damage, and cardiac fibrosis [44]. Furthermore, the MR is also expressed in the endothelium, vascular smooth muscle cells, podocytes, and mesangial cells, favoring the progression of glomerulosclerosis, renal fibrosis, proteinuria, decreased eGFR, and diabetic nephropathy [45]. Several studies in animal models showed that although the MR is essential for normal renal and cardiac functions, its overactivation leads to the production of reactive oxygen species, inflammation, and fibrosis [46,47,48].

With this background, the crucial role of mineralocorticoid receptor antagonists (MRAs) in preventing inflammation and fibrosis in both the kidneys and the cardiovascular system appears clear. Indeed, MRAs represent the first-line therapy in patients with HF [5], and their use has also been associated with reduced proteinuria and a slowed progression of renal failure [49]. MRAs have been designed either with a steroidal or a non-steroidal structure. Steroidal MRAs, including spironolactone, canrenone, and eplerenone, show strong anti-mineralocorticoid, moderate antiandrogen, and weak anti-steroidogenesis effects [12]. Spironolactone, sharing similar structural elements with progesterone, is associated with progestogenic and antiandrogenic adverse effects, including gynecomastia, breast tenderness, and feminization [50]. Conversely, eplerenone is a spironolactone derivative with a higher specificity for the MR, less antiandrogenic and progestogenic side effects, and a shorter plasma half-life compared to spironolactone (4–6 h vs. >12 h) [50]. From a biochemical point of view, spironolactone and eplerenone are “passive” antagonists instead of agonist ligands because they bind the MR without changing the receptor conformation, so transcriptional coregulators can still bind it and permit gene expression [51].

Several studies demonstrated the beneficial effects of steroid MRA treatments in patients with HF, resulting in a significant reduction in mortality and rehospitalizations, and an increase in symptom improvement, without increasing safety events [52,53,54]. Similarly, MRAs could reduce proteinuria and systolic blood pressure in patients with mild to moderate CKD, albeit with an increased risk of hyperkaliemia, acute kidney injury, and gynecomastia [55]. Furthermore, using spironolactone in early-stage CKD significantly improves the LV mass and arterial stiffness [56]. Since steroidal MRAs have a relative or absolute contraindication in end-stage CKD, minimal data exist on their use in this clinical setting. In 2016, a meta-analysis of nine trials showed how the administration of MRAs in 829 patients receiving dialysis reduced cardiovascular mortality by 66%, even though they increased the risk of hyperkaliemia [57]. Moreover, the Aldosterone bloCkade for Health Improvement EValuation in End-stage renal disease (ACHIEVE) trial and the ALdosterone antagonist Chronic HEModialysis Interventional Survival Trial (ALCHEMIST) are two randomized controlled trials that are currently ongoing which will assess the efficacy and safety of spironolactone in patients with end-stage renal disease requiring dialysis [58].

Non-steroidal MRAs, which include apararenone, esaxerenone, and finerenone, are a novel class of drug developed to provide the same efficacy as steroidal MRAs but with fewer side effects, with particular attention to hyperkalemia. Compared to steroidal MRAs, these new molecules show addictive anti-inflammatory and anti-fibrotic effects, as they prevent the transcription of pro-fibrotic and pro-inflammatory genes [51]. Several clinical trials investigating the effects of non-steroidal MRAs are currently ongoing. Indeed, the double-blind randomized phase three study Esaxerenone and Eplerenone in Patients with Essential Hypertension (ESAX-HTN) found that a 5mg daily dose of esaxerenone is effective and well-tolerated in Japanese patients with hypertension, with an antihypertensive effect equivalent to eplerenone [59]. Furthermore, the phase three randomized controlled clinical trial Esaxerenone in Patients with Type 2 Diabetes and Microalbuminuria (ESAX-DN) showed that adding esaxerenone to RAAS inhibitor therapy in patients with DM and microalbuminuria returned albuminuria to normal levels and reduced its progression [60].

## 5. Finerenone: From Biochemical Characteristics to Experimental Studies

**Figure 2:**
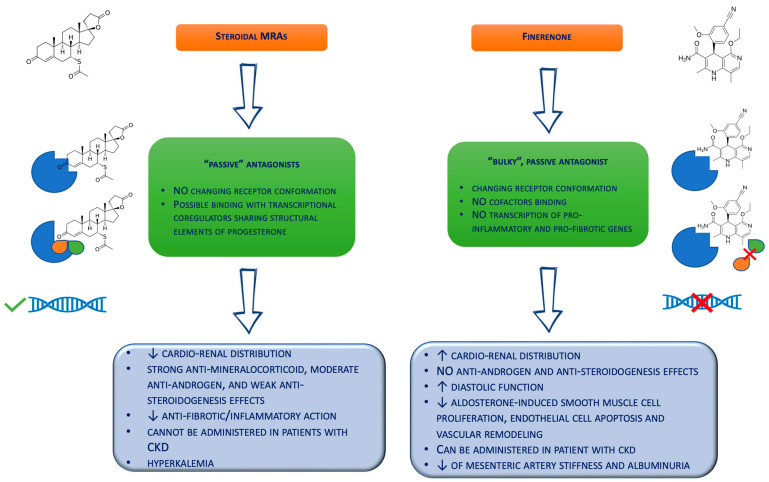
Biochemical differences between the steroidal mineralocorticoid receptor antagonists and finerenone. CKD = chronic kidney disease; MRAs = mineralocorticoid receptor antagonists.

Finerenone belongs to the class of selective nonsteroidal MRAs, exhibiting a higher binding affinity to the mineralocorticoid receptor compared to eplerenone and spironolactone, as well as showing chemical and physical properties that provide a more balanced cardio–renal drug delivery (Figure 2) [16]. Drug metabolism is pre-dominantly hepatic, involving cytochrome P450 3A4 (CYP3A4) and cytochrome P450 2C8 (CYP2C8) [61]. Finerenone could be an inhibitor of CYP2C8 (reversible) and CYP3A4 (reversible and irreversible) and an inducer of CYP3A4 with a 20 mg daily dose, as demonstrated by in vitro experiments with hepatic microsomes and human hepatocytes [62,63]. However, recent studies investigating the once-daily 20 mg administration showed no significant drug–drug interactions with the cytochrome P450 enzyme substrates [64].

Specifically, although a potential clinically relevant induction of CYP3A4 was shown in in vitro experiments, a weak induction was confirmed in vivo [62,64]. In addition, the renal function also influenced the drug clearance. As kidney dysfunction worsened and the clearance decreased, a longer elimination half-life was observed, albeit with no affection on the maximum serum concentration [61]. Furthermore, finerenone shows greater polarity, defined as a higher polar surface area, and is less lipophilic compared to steroidal MRAs [65]. Indeed, finerenone does not cross the blood–brain barrier and preclinical studies demonstrated how this drug was not detected in brain tissue after oral administration [66]. Finally, in hypo-albuminemia, an elevated blood concentration of the drug is observed, as the levels of finerenone are influenced by the serum albumin, which is the main binding protein of this drug [67].

Notably, molecular studies suggest that finerenone acts on the mineralocorticoid receptors as a “bulky” passive antagonist, different from steroidal MRAs [68]. Specifically, this agent works as a bulky substituent of the MR, changing the receptor conformation to avoid binding with cofactors and the transcription of pro-inflammatory and pro-fibrotic genes [69]. Thus, this non-steroidal MRA is able to reduce the MR nuclear accumulation, inhibit the receptor recruitment onto DNA target sequences, and suppress recycling and the induction of mutant forms of the mineralocorticoid receptor [69]. Moreover, finerenone demonstrated a balanced distribution in the cardiac and renal tissues of mice [16], compared to eplerenone and spironolactone, which revealed higher concentrations in the kidney rather than the heart [16]. This combination of the potency and selectivity on the MR and the cardio–renal distribution could translate into expanded protection on the kidneys and heart, particularly in high-risk patients with altered renal functions. In fact, the half-life of finerenone is not affected by mild renal impairment, while it is slightly increased in patients with moderate and severe renal impairment. [61]. However, renal impairment showed no significant effect on the maximum plasma concentration [61]. Moreover, finerenone demonstrated a short half-life in patients with renal failure (about 3 h) and no active metabolites [63]. Conversely, spironolactone metabolites could be measured in approx. 40% of patients with an eGFR of 25–45 mL/min/1.73 m^2^ for up to 3 weeks after stopping the drug administration [70].

Several pre-clinical studies investigated the role of finerenone on cardio-renal diseases (Table 1).

Firstly, Bonnard et al. showed that in mice with CKD induced using subtotal nephrectomy, treatment with finerenone improved the diastolic function, which was severely impaired due to cardiac hypertrophy and fibrosis [71]. Finerenone also reduced the plasmatic levels of the prohormone of the brain natriuretic peptide [16].

Beyond its benefits on cardiac remodeling, finerenone showed favorable effects on the vascular system. This agent dose-dependently reduced the aldosterone-induced smooth muscle cell proliferation and endothelial cells apoptosis [72]. Furthermore, it was able to accelerate re-endothelialization and inhibit vascular remodeling after vascular damage [72]. In addition, in mice with predefined CKD, finerenone could improve endothelial dysfunction through an increased nitric oxide (NO) bioavailability and reduced levels of superoxide anion levels [73], thus decreasing the oxidative stress and its negative effects on the cardiovascular system [15]. Interestingly, the administration of finerenone improved metabolic syndrome-related disorders, such as diastolic cardiac dysfunction and nephropathy, in rats with metabolic syndromes. This benefit was mediated by short-term effects, such as improving myocardial perfusion and decreasing the oxidative stress, and long-term effects, such as reducing the LV diameters and LV end-diastolic pressure [74]. Moreover, finerenone was associated with reduced mesenteric artery stiffness and albuminuria in animal models, which were directly related to CKD and increased cardiovascular morbidity and mortality [75]. Furthermore, as shown by Kolkhof et al. [16], finerenone could induce end-organ protection with a decreased risk of electrolyte disturbances.

## 6. Finerenone: From Bench to Bedside

Clinical trials which evaluated the role of finerenone on cardiovascular outcomes were recently published, and several are still ongoing (Table 2) [68].

In the phase II Mineralocorticoid Receptor Antagonist Tolerability Study (ARTS) trial, the authors demonstrated a similar efficacy of the daily finerenone treatment (5 or 10 mg) compared to spironolactone in patients suffering from HF with a reduced ejection fraction and mild CKD and with lower increases in the serum potassium levels and slight reduction in the eGFR [66]. Specifically, the administration of finerenone was associated with significantly smaller mean increases in the potassium levels compared to spironolactone (0.04–0.30 and 0.45 mmol/L, respectively, *p* < 0.0001–0.0107) and reduced rates of hyperkalemia (5.3 and 12.7%, respectively, *p* = 0.048) [66].

Additionally, the phase IIb ARTS-Diabetic Nephropathy (ARTS-DN) trial showed the efficacy and safety of finerenone in patients with DM and high or very high proteinuria due to an improvement in the urinary albumin–creatinine ratio with no difference in the rate of hyperkalemia and the eGFR reduction compared to the placebo [76]. Concordantly, the Mineralocorticoid Receptor Antagonist Tolerability Study-Heart Failure (ARTS-HF) was designed to compare the efficacy and safety of finerenone with eplerenone in patients with type 2 DM and/or CKD suffering from chronic HF with a reduced ejection fraction and already treated with evidence-based therapy for HF for at least three months [77]. Specifically, the study design consisted of five pre-planned finerenone treatment arms and one current eplerenone treatment. The primary aim was to examinate the efficacy and safety of different oral doses of finerenone given once per day [77]. While the primary endpoint of a 30% reduction in NT-proBNP level after 90 days was not achieved (with similar results in the two groups), the incidence of the exploratory composite endpoint of death from any cause, cardiovascular hospitalization, or emergency presentation for worsening HF at day 90 was significantly lower in most finerenone groups (especially the 10 titrated up to 20 mg dose group) compared to eplerenone [77]. Notably, an increase in the serum potassium levels above ≥5.6 mmol/L at any time was observed in approx. 4% of patients, with a similar distribution among all the treatment groups [77].

The two largest trials that explored the clinical benefit of finerenone on the renal and cardiovascular outcomes in patients with mild to severe CKD and type 2 DM, on top of the maximum tolerated renin–angiotensin system inhibition, were the Finerenone in Reducing Kidney Failure and Disease Progression in Diabetic Kidney Disease (FIDELIO-DKD) [17] and the Finerenone in Reducing Cardiovascular Mortality and Morbidity in Diabetic Kidney Disease (FIGARO-DKD) trials [18].

FIDELIO-DKD was a double-blind, placebo-controlled trial in which the patients were randomized 1:1 to finerenone (10 or 20 mg) or placebo treatments. Different from the previous studies, the patients with previous HF with a reduced ejection fraction were excluded from the study [17]. Chronic kidney disease was defined as either persistent, moderately increased albuminuria (urinary albumin-to-creatinine ratio [UACR] ≥30–<300 mg/g) with an eGFR of ≥25–<60 mL/min/1.73 m^2^ and the presence of diabetic retinopathy, or persistent, severely increased albuminuria (UACR ≥300–≤5000 mg/g) with an eGFR of ≥25–<75 mL/min/1.73 m^2^ [17]. The cardiovascular outcome was a composite of the time to the first onset of cardiovascular death, non-fatal myocardial infarction, non-fatal stroke, or hospitalization for HF. The kidney outcome was a composite of kidney failure (defined as chronic dialysis for >90 days, kidney transplantation, or eGFR <15 mL per min per 1.73 m^2^), a sustained ≥40% decrease in the eGFR from the baseline over at least four weeks, or renal death [17]. Otherwise, the secondary cardiac outcome consisted of a composite of death from CV causes, non-fatal myocardial infarction, non-fatal stroke, or hospitalization for HF, with a time-to-event analysis. The results demonstrated a lower incidence of the composite cardiovascular outcome in the finerenone group than in the placebo group (367 [13.0%] and 420 [14.8%] patients, respectively; [95% CI, 0.75–0.99]; *p* = 0.034) regardless of the presence of previous CVD.

A lower incidence of the composite renal outcome was also achieved in the finerenone-treated group compared to the placebo (200 [15.3%] vs. 267 [20.5%], respectively), particularly in the patients with CVD [17]. In addition, Filippatos et al. published a secondary analysis of the FIDELIO-DKD trial analyzing the effects of the novel MRA on the incidence of new onset atrial fibrillation/atrial flutter in the same cohort of patients [78]. Interestingly, in the group of patients randomized for the finerenone treatment, there was a reduction in the absolute risk of new onset atrial arrhythmias and a lower incidence of renal or cardiovascular events regardless of a history of atrial fibrillation/flutter at the baseline [78].

The FIGARO-DKD was a randomized, double-blind, placebo-controlled study where patients with type 2 diabetes and CKD were randomly assigned for finerenone treatment or placebo [18]. Chronic kidney disease in FIGARO-DKD was defined as either persistent, moderately increased albuminuria (UACR ≥ 30–<300 mg/g) with an eGFR of ≥25–≤90 mL/min/1.73 m^2^, or persistent, severely increased albuminuria (UACR ≥ 300–≤ 5000 mg/g) with an eGFR ≥ 60 mL/min/1.73 m^2^ [18]. The primary outcome was a composite of death from CV causes, non-fatal myocardial infarction, non-fatal stroke, or hospitalization for HF. The secondary outcome was a composite of kidney failure (defined as chronic dialysis for >90 days, kidney transplantation, or eGFR < 15 mL per min per 1.73 m^2^ for at least four weeks), a sustained decrease from the baseline of at least 40% in the eGFR, or death from renal causes [18]. The primary outcome occurred in 12.4% of the patients treated with the novel MRA, compared to 14.2% in the placebo group (*p* = 0.03). The secondary outcome occurred in 9.5% of the treatment group vs. 10.8% of the placebo-treated patients [18]. Remarkably, this study demonstrated a significant reduction in cardiovascular outcome in the finerenone group, with the major contribution driven by a lower incidence of hospitalization and a reduction in the secondary kidney outcome, albeit not significant [18]. Interestingly, a study on the combined effect of finerenone and empagliflozin in patients with type 2 diabetes and CKD is still ongoing and is estimated to be completed by 2024 [79]. This study is designed to evaluate the cumulative efficacy, safety, and tolerability of dual therapy, including finerenone and empagliflozin in people with CKD and diabetes [79]. Considering the well-known protective effects of sodium–glucose transporter 2 inhibitors (SGLT2i) on the cardiovascular system [80] and renal functions [9], dual therapy with finerenone and SGLT2i could provide an additive effect in order to slow the disease progression and provide long-term benefits for patients with diabetes and CKD [79].

Based on the above trials, both the U.S. Food and Drug Administration (FDA) and the European Medicines Agency (EMA) approved the marketing of finerenone in routine clinical care for patients with CKD and DM, although studies are currently ongoing to formulate economic models to evaluate its use. Indeed, drug utilization in the real world is unequal from nation to nation and is influenced by drug reimbursability ($22.13 per unit in USA), local care factors, and standard medical therapy. The ongoing FINE REAL study aims to evaluate the use of finerenone in a variety of states by relating it to specific routine clinical care, and by assessing its impact in terms of its improvement in the progression of microvascular complications and renal functions [81].

## 7. Strengths and Limitations

This comprehensive review aimed to summarize the current knowledge regarding finerenone and its effects on chronic kidney disease and the cardiovascular system. As CKD and CVD are strictly related, we firstly analyzed this association to explain the worse CV outcomes in patients with CKD compared to those with normal renal functions. Secondly, we evaluated the crucial role of the RAAS and mineralocorticoid overactivation, which negatively affect both the renal and cardiovascular systems, favoring inflammation, oxidative stress, and fibrosis, and subsequently the progression of kidney and heart failure. Notably, we strongly believe that a deep knowledge of pathophysiological mechanisms is essential to understand the beneficial effects of this novel drug on the cardiorenal system.

Nevertheless, this study had several limitations. Firstly, due to its nature, this non-systematic review was not conducted according to a specific protocol and/or guidelines, thus influencing the quality and significance of the data reported. Secondly, this review was carried out while different clinical trials were ongoing, so we could provide only preliminary results. Lastly, considering the novelty of this drug, further studies are needed in order to evaluate its efficacy and safety in the long term.

## 8. Conclusions

Patients with chronic kidney disease show a high cardiovascular risk, with cardiovascular death as the main cause of death. Finerenone administration in diabetic patients with CKD could reduce the overall cardiovascular risk, independently from the presence of the baseline cardiovascular disease. Indeed, patients with CKD and type 2 diabetes treated with finerenone showed a lower incidence of cardiovascular death, non-fatal myocardial infarction, and hospitalization for heart failure compared to the placebo. Moreover, due to its pharmacokinetic profile, this novel drug did not induce antiandrogen and anti-steroidogenesis effects. In addition, compared to spironolactone, finerenone induced smaller mean increases in potassium levels and reduced rates of hyperkalemia.

In conclusion, finerenone confers significant renal and cardiovascular benefits in patients with CKD, resulting in effectively slowing the progression of kidney failure in patients with CKD and reducing the risk of major adverse CV events in this population, thus playing a crucial role in the cardiorenal system.

## 9. Future Directions

As discussed above, finerenone represents a novel promising therapeutic agent for patients with CKD, conferring significant renal and cardiovascular benefits. Although still not approved for the use in a heart failure context, finerenone could play a crucial role in the management of HF patients, taking into account how patients with impaired renal function cannot benefit from optimized cardiovascular medical therapy. However, further studies and clinical trials are needed to evaluate the efficacy and safety of finerenone in patients with CKD and CVD, considering the possible interaction and synergic effects of new therapeutic strategies (i.e., sodium–glucose cotransporter 2 inhibitors).

## Figures and Tables

**Table 1:** Results of pre-clinical studies evaluating the role of finerenone on cardio-renal system.

Study	Setting	Results
Kolkhof et al., 2014 [16]	Sprague-Dawley rats and Wistar rats (two different preclinical rat disease models)	↓ Cardiac hypertrophy ↓ Plasma prohormone of the brain natriuretic peptide ↓ Proteinuria ↑ Systolic and diastolic left ventricular function ↑ End-organ protection ↓ Risk of electrolyte disturbances
Dutzmann et al., 2017 [41]	Human coronary artery SMC and human umbilical vein ECs	↓ Apoptosis of ECs ↓ SMC proliferation ↑ Endothelial healing ↓ Neointima formation of the injured vessels
Bonnard et al., 2018 [42]	Mice with CKD induced through subtotal nephrectomy	↑ In systolic and diastolic cardiac function ↑ LV contractility (↑ LV end-systolic pressure–volume relationship-LVESPR) ↓ LV end-diastolic pressure–volume relationship-LVEDPR ↓ (LVEDP) ↓ LV hypertrophy and fibrosis
González-Bláquez et al., 2018 [43]	Munich Wistar Frömter rats (a genetic model of chronic kidney disease)	↓ Endothelial dysfunction ↑ NO bioavailability ↓ Superoxide anion levels ↑ SOD activity ↓ Albuminuria
Lachaux et al., 2018 [44]	Zucker fa/fa rats (a rat model of metabolic syndrome)	Long-term effects =Blood pressure and heart rate ↓ LV end-diastolic pressure and LV end-diastolic pressure–volume relationship =LV end-systolic pressure and LV end-systolic pressure–volume relationship ↓ LV systolic and diastolic diameters ↓ LV weight and LV collagen density ↓ Proteinuria and renal nGAL expression Short-term effects ↓ LV systolic diameter =LV diastolic diameter ↑ Myocardial tissue perfusion ↓ Myocardial reactive oxygen species ↑ Plasma nitrite levels (NO bioavailability)
Gil-Ortega et al., 2020 [45]	Munich Wistar Frömter rats (a genetic model of chronic kidney disease)	↓ Intrinsic (mesenteric) arterial stiffness ↓ Albuminuria ↑ Plasma pro-MMP-2 activity ↓ Plasma MMP-2 and MMP-9 activities ↑ NO bioavailability ↓Superoxide anion levels

Abbreviations: CKD = chronic kidney disease; ECs = endothelias cells; LV = left ventricle; LVEDP = left ventricle end-diastolic pressure; LVEDPR = left ventricle end-diastolic pressure–volume relationship; LVESPR = left ventricle end-systolic pressure–volume relationship; MMP = matrix metalloproteinases; nGAL = neutrophil gelatinase-associated lipocalin; NO = nitric oxide; SMC = smooth muscle cell; SOD = superoxide dismutase; ↑ = raised; ↓ = reduced.

**Table 2:** Results of clinical trials evaluating the role of finerenone on cardiovascular outcomes.

	Patients	Primary Outcome	Secondary Outcomes	Results
ARTS (Part A: finerenone vs. placebo; part B: finerenone vs. spironolactone or placebo) 2012 [46]	65 (part A); 392 (part B)	Change in the serum potassium concentration vs. placebo	(i) Changes in the serum potassium concentration vs. spironolactone (ii) Changes in the biomarkers of the cardiac and renal function or injury, eGFR (MDRD), and albuminuria	- Similar efficacy - Smaller increases the in serum potassium concentration
ARTS-DN (finerenone vs. placebo) 2015 [47]	823	Ratio of the urinary albumin–creatinine ratio (UACR) at day 90 vs. at the baseline	(i) Adverse and serious adverse events (ii) Changes in the serum potassium levels (iii) Incidence of a decrease in eGFR of 30% or more, 40% or more, and 57% or more (iv) Changes in the UACR at day 30 and day 60 relative to the baseline	- Improvement in the UACR - No occurrences of eGFR decreases of at least 57% - No difference in the overall incidence of adverse events - Only a modest reduction in blood pressure at the highest dosage of finerenone
ARTS-HF (finerenone vs. eplerone) 2016 [48]	1055	Efficacy (rate of patients who had a 30% reduction in the NT-proBNP level after 90 days) and safety (i.e., serum potassium concentration, vital signs, biomarkers of organ injury,...)	(i) Composite endpoint of death from any cause, cardiovascular hospitalization, or emergency presentation for worsening chronic HF until day 90 (ii) Change in efficacy biomarkers (BNP, NT-proBNP, galectin 3, and N-terminal procollagen III propeptide) (iii) Change in the scores on health-related quality of life (QoL) questionnaires [the Kansas City Cardiomyopathy Questionnaire (KCCQ) and the five-dimension European Quality of Life Questionnaire (EuroQoL).	- Similar efficacy - Reduction in the composite endpoint of death from any cause, cardiovascular hospitalization, or emergency presentation for worsening chronic HF until day 90 - Similar increase in the serum potassium concentration - Similar changes in the questionnaire mean scores.
FIDELIO-DKD (finerenone vs. placebo) 2020 [17]	5674	Composite of kidney failure, a sustained decrease of at least 40% in the eGFR from the baseline over a period of at least 4 weeks, or death from renal causes (time-to-event analysis).	(i) Composite of death from cardiovascular causes, nonfatal myocardial infarction, nonfatal stroke, or hospitalization for heart failure (ii) Death from any cause (iii) Hospitalization for any cause (iv) Change in the UACR from the baseline to month 4 (v) Composite of kidney failure, a sustained decrease of at least 57% in the eGFR from the baseline, or death from renal causes	- Significant reduction in the primary composite outcome - Significantly lower risk of a key secondary outcome event - Similar risk of nonfatal stroke.
FIGARO-DKD (finerenone vs. placebo) 2021 [18]	7352	Composite of death from cardiovascular causes, nonfatal myocardial infarction, nonfatal stroke, or hospitalization for heart failure (time-to-event analysis).	(i) Composite of the first occurrence of kidney failure, a sustained decrease from the baseline of at least 40% in the eGFR for a period of at least 4 weeks, or death from renal causes (ii) Hospitalization for any cause (iii) Death from any cause (iv) Change in the UACR from the baseline to month four (v) Kidney composite outcome of the first onset of kidney failure, a sustained decrease from baseline of at least 57% in the eGFR for a period of at least 4 weeks, or death from renal causes	- Significantly lower incidence of the primary composite outcome - Lower incidence of hospitalization for any cause and death for any cause. - No significant between-group difference in the incidence of the first or secondary composite outcome. - Greater reduction in the UACR from the baseline to month four. - Lower incidence of a secondary kidney composite outcome

Abbreviations: BNP = natriuretic peptide; eGFR = estimated glomerular filtration rate; HF = heart failure; MDRD = modification of diet in renal disease; NT pro-BNP = N-terminal pro-brain natriuretic peptide; UACR = urinary albumin–creatinine ratio.

## Data Availability

Not applicable.

## Appendix A

As mentioned in the Methods section, we conducted this review using a literature search of the PubMed, EBSCO Embase, and Cochrane databases of systematic reviews. An example of a search string used on Pubmed was “(finerenone) AND (cardiovascular disease)) AND (chronic kidney disease)”.
